# The role of coagulation parameters in the assessment of acute pancreatitis severity: A retrospective study

**DOI:** 10.1097/MD.0000000000049392

**Published:** 2026-06-19

**Authors:** Hongda Zhu, Mengqi Hong, Wenyi Hu, Qiyan Yu

**Affiliations:** a Department of Hepato-Pancreato-Billiary Surgery, The Affiliated Lihuili Hospital of Ningbo University, Ningbo, Zhejiang, China; b Department of Emergency, Ningbo Ninth Hospital, Ningbo, Zhejiang, China.

**Keywords:** assessment indicators, bioinformatics analysis, coagulation function indicators, MAP, SAP

## Abstract

Acute pancreatitis (AP) is a common and rapidly progressing disease of the digestive system. The occurrence of coagulation dysfunction is closely related to the severity of AP. Therefore, this study aims to explore the correlation and mechanism of early clinical coagulation function indicators in AP patients with the severity of the condition. Between January 2021 and May 2023, 60 cases of AP patients were divided into 2 groups: mild AP and severe AP. Coagulation parameters, including prothrombin time (PT), activated partial thromboplastin time, fibrinogen (FIB), and D-dimer (DD) levels, were measured. The Acute Physiology and Chronic Health Evaluation II score, Ranson score, and computerized tomography severity index score were used for Spearman linear correlation analysis of PT, activated partial thromboplastin time, FIB, and DD. Multifactor logistic stepwise regression analysis was conducted to identify the risk factors influencing the severity of AP. Receiver operating characteristic curve analysis was used to evaluate the diagnostic value of various indicators between the severe AP group and the mild AP group. Through bioinformatics methods, genes with significant differential expression and the pathophysiological processes associated with these genes were analyzed to explore the potential mechanisms and key genes involved in the progression of AP. PT > 12.2 seconds, FIB > 3.124 g/L, and DD > 0.97 mg/L are significantly correlated with the severity of AP and can serve as valuable indicators for assessing disease severity. Their potential mechanism may involve the CD177 and ALOX15/15-HETE signaling pathway, which affects the occurrence and development of AP by regulating coagulation function.

## 1. Introduction

Acute pancreatitis (AP) is a common disease of the digestive system characterized by rapid onset and progression. When the disease progresses to severe pancreatitis, it not only causes severe damage to the structure and function of the gastrointestinal mucosa but also leads to the entry of inflammatory mediators, intestinal flora, endotoxins, and other substances into the bloodstream. This can trigger systemic inflammatory response syndrome and multiple organ dysfunction syndrome, serious complications that result in poor patient outcomes.^[1,2]^ Therefore, accurately assessing the patient’s condition is of great value in both clinical decision-making for appropriate treatment measures and reducing the mortality rate of patients.

Currently, clinical assessment schemes for pancreatitis patients include the Ranson criteria, Acute Physiology and Chronic Health Evaluation II (APACHE II), and CT severity index (CTSI). However, these indicators still lack dynamic monitoring of the progression of AP, the relationship between disease severity, and the body’s coagulation function.^[3,4]^ Therefore, finding new dynamic indicators to evaluate the onset, progression, and development of AP could lead to a more scientific and effective evaluation of disease severity, providing better treatment options, and improving patient survival rates.

Previous studies have indicated that the onset and progression of AP are typically accompanied by a series of pathological processes, including platelet aggregation activation, increased vascular permeability, systemic changes in the coagulation and fibrinogen (FIB) systems, pancreatic microcirculation disorders, and alterations in platelet parameters.^[5–7]^ Prothrombin time (PT) serves as a sensitive indicator of the exogenous coagulation system, while activated partial thromboplastin time (APTT) is a sensitive indicator of the endogenous coagulation system. FIB is an acutely responsive protein with coagulation function primarily synthesized by liver cells. Under the influence of thrombin and calcium ions, it can form stable insoluble fibrin clots, playing a crucial role in thrombus formation.^[8]^ D-dimer (DD) functions as a sensitive indicator of abnormalities in the coagulation and FIB systems, representing degradation products of fibrin. Elevated DD levels indicate a hypercoagulable state and secondary activation of the FIB system.^[9]^

A recent study discovered abnormalities in the coagulation and FIB systems in patients with hypertriglyceridemia-induced AP at an early stage. PT, FIB, and DD levels increase with the severity of AP and are positively correlated with it.^[10]^ However, despite being readily available indicators that can be obtained early and effectively, whether coagulation function is significantly correlated with the severity of AP in patients early on and its underlying mechanisms have not been reported in relevant studies to date. Therefore, this study aims to focus on the early detection of coagulation function indicators PT, APTT, FIB, and DD in the initial stages of AP onset, exploring the application value of coagulation function indicators in the early diagnosis of severe AP (SAP) and mild AP (MAP) and their correlation with disease severity.

Moreover, through bioinformatics analysis, this study explores the mechanisms and pathways in which differentially expressed genes (DEGs) associated with coagulation factors participate in AP. This provides a theoretical basis for utilizing coagulation function indicators to assess the severity of AP and guide anticoagulant therapy.

## 2. Materials and methods

### 2.1. General information

This study is a single-center retrospective cohort study that collected data from patients admitted with AP between January 2021 and May 2023. All subjects experienced their first onset of the disease upon admission. A total of 60 patients met the inclusion criteria. The inclusion criteria were as follows: age ≥18 years, diagnosis of AP according to the diagnostic criteria (reference), time interval from onset to admission ≤72 hours, first admission for AP, and patient or family consent to participate in the study and signed informed consent. The exclusion criteria were as follows: primary liver dysfunction; use of any procoagulant or anticoagulant drugs within 7 days; severe hematological disorders affecting coagulation function, such as aplastic anemia; presence of malignant tumors; and pregnant or lactating women. Dropout criteria during the study were defined as follows: death of the subject during the study; subjects unwilling to continue participating in the study; and other reasons preventing subjects from continuing in the study. Ethical approval for this study was granted by the Ningbo Ninth Hospital Medical Health Group Ethics Committee (Approval Number: 2024LIK11), and written informed consent was obtained from all participants.

Based on the patients’ clinical characteristics, laboratory test results, imaging findings, and clinical diagnoses following the revised Atlanta classification criteria from 2012,^[11]^ the patients were divided into 2 groups: the MAP group and the SAP group.

### 2.2. Observation indicators

Measurement and evaluation indicators: both the MAP group and SAP group had peripheral blood samples collected within 24 hours of admission. The immunoturbidimetric method was used to measure the levels of PT, APTT, FI, and DD in the peripheral blood of each study subject using the CA-500 fully automatic coagulation analyzer. The study subjects were also assessed using the APACHE II score^[12]^ and Ranson score^[13]^ upon admission. All study subjects underwent a computerized tomography (CT) scan on the first day of admission, and the images were processed, read, and evaluated for the Balthazar CTSI by 2 experienced radiologists. The evaluation method involved combining the Balthazar CT grading standard with the extent of pancreatic necrosis.^[14]^ The Balthazar CT grading ranged from grades A to E, with corresponding scores of 0 to 4. Additional points were added based on the percentage of pancreatic necrosis: <30% added 2 points, 30% to 50% added 4 points, and over 50% added 6 points. The severity of SAP was classified into 3 levels based on the total score: 1st level for scores 0 to 3, 2nd level for scores 4 to 6, and 3rd level for scores 7 to 10.

### 2.3. Bioinformatics analyses

#### 2.3.1. Gene Expression Omnibus data

From the Gene Expression Omnibus database, we selected RNA sequencing samples from the peripheral whole blood of patients with MAP, moderate AP, and SAP (GSE194331, n = 87). Volcano plot analysis was performed for all DEGs, with log2(fold change) as the x-axis and −log10(*P*-value) as the y-axis. DEGs meeting the criteria of log2(fold change) < −1 or >1 and −log10(*P*-value) > 1 were included in this study. The volcano plot was used to visualize the distribution of DEGs, where green represents significantly downregulated DEGs, red represents upregulated DEGs, and gray indicates genes without statistical significance.

#### 2.3.2. R software

Data were analyzed using R (version 3.6.3; statistical analysis and visualization, RStudio integrated development environment [IDE]), Gene ontology/Kyoto Encyclopedia of Genes and Genomes (KEGG) enrichment analysis was performed using the Cluster-Profiler package (version 3.18.0). The ggplot2 software package was used to analyze the data. LogFC > 2 and *P* value < .01 were set as the thresholds for statistical difference. The results of the differential analysis are shown using volcano and heat maps.

### 2.4. Statistical method

Statistical analysis was performed using SPSS 25.0 software (IBM Corp.). For data analysis, normally distributed data were expressed as mean ± standard deviation, while skewed data were represented using the median and interquartile range. Group comparisons of normally distributed data were conducted using the *t* test, while skewed data comparisons used the Mann–Whitney *U* test. The Spearman linear correlation analysis was employed to evaluate the relationships between the APACHE II score, Ranson score, CTSI score, and PT, APTT, FIB, and DD levels, with the strength of the relationships indicated by the correlation coefficient “*r*.” Multiple logistic regression analysis was utilized to identify risk factors influencing the severity of AP. Receiver operating characteristic (ROC) curve analysis was performed, and the area under the curve was calculated to assess the diagnostic value of coagulation function indicators in evaluating the severity of early-stage SAP and MAP. A significance level of *P* < .05 was set to indicate statistical differences.

## 3. Results

### 3.1. Comparison of baseline data, coagulation function indicators, APACHE II score, Ranson score, and CTSI score between the MAP group and SAP group

Table 1 displays the data comparison between the MAP group and SAP group, comprising a total of 60 patients. The MAP group consisted of 99 individuals, including 57 males and 42 females, while the SAP group comprised 81 individuals, with 51 males and 30 females. In the MAP group, the age ranged from 18 to 68 years, with an average of 54.12 ± 15.95 years, and the blood collection time ranged from 4.3 to 11.5 hours, averaging 9.36 ± 2.17 hours. For the SAP group, the age range was 19 to 67 years, with an average of 55.22 ± 16.61 years, and blood collection time ranged from 4.1 to 11.9 hours, averaging 9.28 ± 2.24 hours. There were no statistically significant differences in gender, age, and disease duration between the MAP and SAP groups (*P* > .05).

**Table 1:** Clinical characteristics and comparison of coagulating function indexes, APACHE II, Ranson, and CTSI scores in patients in the MAP group and SAP group.

Variables	MAP group	SAP group	*t*/χ^*2*^/*z*	*P*
Sex (male), n (%)	57 (57.58)	51 (62.96)	0.539	.463
Sex (female), n (%)	42 (42.42)	30 (37.04)		
Age, y	54.12 ± 15.95	55.22 ± 16.61	−0.261	.795
Blood draw time, h	9.36 ± 2.17	9.28 ± 2.24	0.639	.264
APTT (s)	25.76 ± 3.66	26.65 ± 3.67	−0.743	.457
PT (s)	11.88 ± 0.91	13.08 ± 1.64	−3.049	.002
Fib (g/L)	2.99 ± 1.16	4.84 ± 5.57	−2.274	.023
DD (mg/L)	1.24 ± 1.08	3.84 ± 5.79	−4.027	.000
APACHE II score	6.16 ± 1.24	10.96 ± 2.17	15.492	.000
Ranson score	4.15 ± 1.17	7.28 ± 2.04	9.648	.021
CTSI score	5.74 ± 1.38	9.83 ± 2.15	10.046	.005

Data are reported as mean and SD, except for gender. *T*-test (t) or chi-squared (χ^2^) or *Z*-test (z) was used.

APACHE II = Acute Physiology and Chronic Health Evaluation II, APTT = activated partial thromboplastin time, CTSI = CT severity index, DD = D-dimer, Fib = fibrinogen, PT = prothrombin time, SD = standard deviation.

The APTT in the MAP group was 25.76 ± 3.66 seconds, while in the SAP group, it was 26.65 ± 3.67 seconds, showing no statistically significant difference (*P* > .05). However, PT, FIB, and DD values were significantly higher in the SAP group compared with the MAP group, with statistically significant differences (*P* < .05). Evaluation of the APACHE II score, Ranson score, and CTSI score within 24 hours of admission revealed significantly higher scores in the SAP group compared with the MAP group, with statistically significant differences observed (*P* < .05).

### 3.2. Correlation analysis between APACHE II score, Ranson score, CTSI score, and various coagulation function indicators

Table 2 illustrates the correlation analysis results. The APACHE II score demonstrates a positive correlation with PT, FIB, and DD but does not correlate with APTT (*P* > .05). Similarly, the Ranson score exhibits a positive correlation with PT, FIB, and DD but shows no correlation with APTT (*P* > .05). Furthermore, the CTSI score displays a positive correlation solely with PT and lacks correlation with APTT, FIB, and DD (*P* > .05).

**Table 2:** Correlation between APACHE II, Ranson, CTSI, and coagulating function indexes.

Variables	Statistics	APTT (s)	PT (s)	Fib (g/L)	DD (mg/L)
APACHE II score	*r*	0.142	0.409	0.272	0.403
	*P*	.280	.001	.035	.001
Ranson score	*r*	0.097	0.397	0.296	0.524
	*P*	.462	.002	.022	.000
CTSI score	*r*	−0.083	0.258	−0.03	0.131
	*P*	.529	.047	.819	.318

APACHE II = Acute Physiology and Chronic Health Evaluation II, APTT = activated partial thromboplastin time, CTSI = CT severity index, DD = D-dimer, Fib = fibrinogen, PT = prothrombin time.

### 3.3. Multifactor logistic regression analysis of AP severity

Upon assigning values to variables, the outcomes outlined in Table 3 were obtained. Subsequently, in Table 4, variables were integrated into a multifactor logistic regression equation, and the final variables incorporated in the equation were PT and DD. The odds ratios for PT and DD were determined to be 3.456 and 2.197, respectively, with statistical significance at *P* < .05. These results signify that with other variables held constant, a unit increase in PT and DD escalates the risk of SAP occurrence by 3.456 times and 2.197 times, respectively.

**Table 3:** Variable assignment.

Factor	Value and meaning
Group	MAP = 1, SAP = 2
Sex	Male = 1, female = 2
APACHE II score	<8 = 1, ≥8 = 2
Ranson score	<3 = 1, ≥3 = 2
CTSI score	I = 1, II = 2, III = 3

APACHE II = Acute Physiology and Chronic Health Evaluation II, CTSI = CT severity index, MAP = mild acute pancreatitis, SAP = severe acute pancreatitis.

**Table 4:** Multivariate logistic regression analysis of the severity of acute pancreatitis.

Variables	Partial regression coefficients	SE	Wald	*P* value	OR	95% CI	
						Lower	Upper
Sex	−0.001	0.702	0.000	.999	0.999	0.253	3.954
Age	−0.025	0.024	1.066	.302	0.976	0.931	1.022
APTT (s)	−0.074	0.100	0.547	.460	0.929	0.764	1.130
PT (s)	0.376	0.300	1.572	.010	3.456	2.809	5.621
Fib (g/L)	0.427	0.262	2.658	.103	1.532	0.917	2.559
DD (mg/L)	0.787	0.309	6.498	.011	2.197	1.200	4.025
Constant	−4.544	4.019	1.278	.258	0.011	–	–

APTT = activated partial thromboplastin time, CI = confidence interval, DD = D-dimer, Fib = fibrinogen, OR = odds ratio, PT = prothrombin time, SE = standard error.

### 3.4. ROC curve analysis of PT, FIB, and DD in the MAP group and SAP group

**Figure 1.**
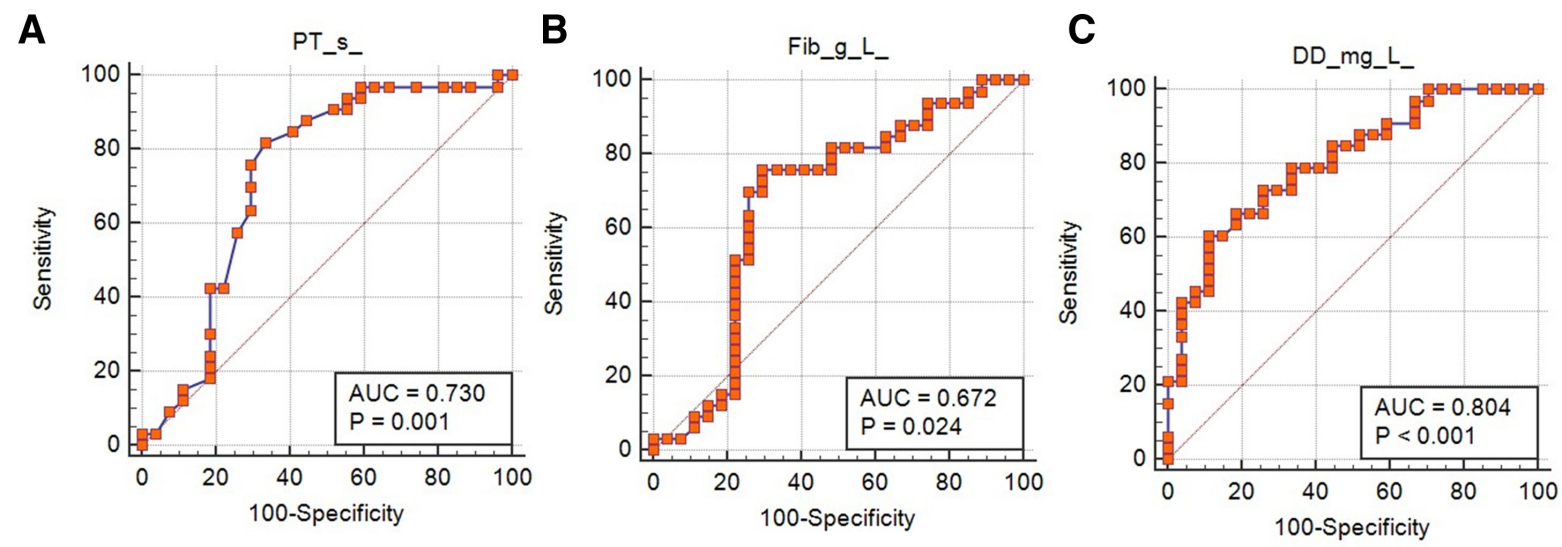
ROC curve of PT, Fib, and DD in the diagnosis of SAP. AUC = area under curve, DD = D-dimer, Fib = fibrinogen, PT = prothrombin time, ROC = receiver operating characteristic.

Figure 1 displays the results of the ROC curve analysis conducted to evaluate the assessment value of PT, FIB, and DD between the SAP group and MAP group. The area under the curve (AUC) for PT was 0.730 with statistical significance at *P* < .05 and a cutoff value of 12.2. Similarly, the AUC for DD was 0.804 with *P* < .05 and a cutoff value of 0.97. In addition, the AUC for FIB was 0.672 with *P* < .05 and a cutoff value of 3.124. These findings suggest that PT > 12.2, FIB > 3.124, and DD > 0.97 demonstrate significant correlations with SAP and can act as early assessment indicators. Consequently, when PT > 12.2, FIB > 3.124, and DD > 0.97, there is an increased likelihood of patients developing SAP, making these indicators valuable as markers for the severity assessment of AP.

### 3.5. Typical case analysis

**Figure 2.**
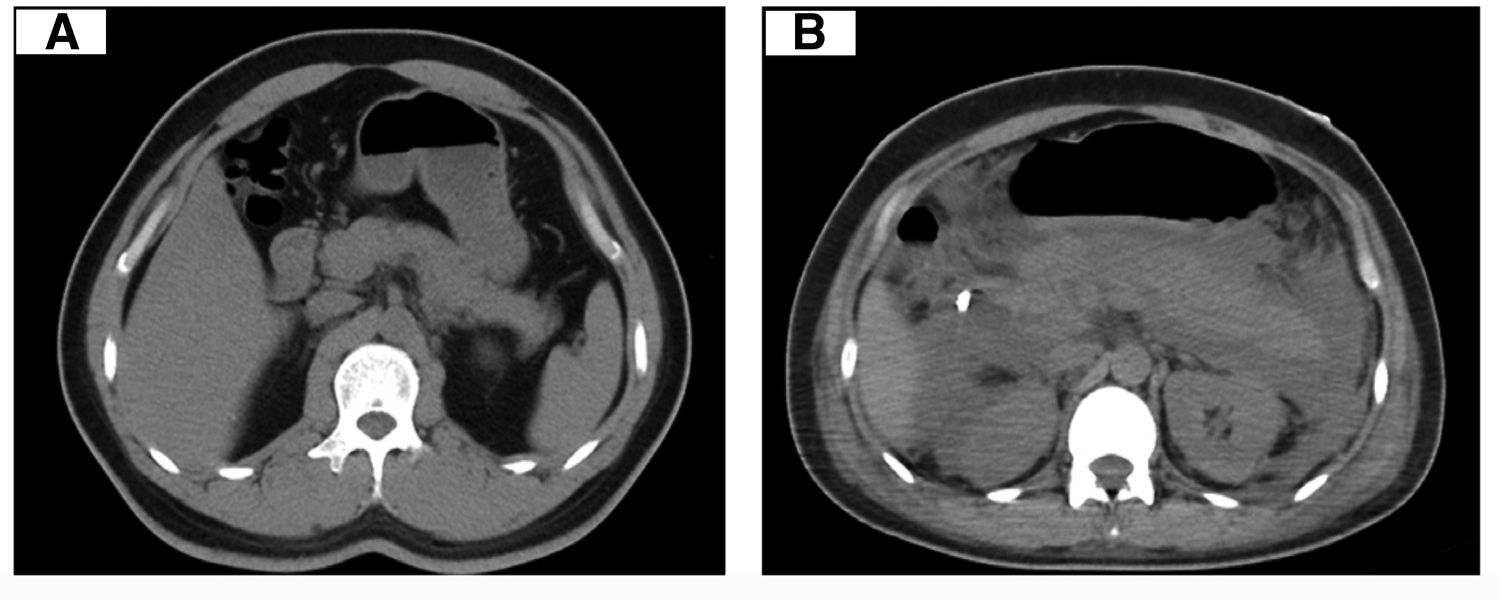
CT characteristics in MAP and SAP: (A) MAP, (B) SAP. CT = computerized tomography, MAP = mild acute pancreatitis, SAP = severe acute pancreatitis.

Figure 2A depicts the CT image of MAP. A 47-year-old male presented with APTT of 25.8 seconds, PT of 11 seconds, FIB level of 2.513g/L, DD level of 0.15 mg/L, APACHE II score < 8, and Ranson score < 3. The CT plain scan reveals the dimensions, morphology, and density of the pancreas, indicating reduced density and slightly indistinct fat interspace around the pancreatic body.

In Figure 2B, the CT image of SAP is illustrated. A 48-year-old male exhibited an APTT of 31.5 seconds, PT of 17.2 seconds, FIB level of 5.308 g/L, DD level of 5.09 mg/L, APACHE II score > 8, and Ranson score ≥ 3. The CT plain scan exhibits pancreatic enlargement, decreased density, serrated pancreatic margins, and a substantial accumulation of fluid within the pancreatic and adjacent organ interspaces.

### 3.6. Analysis of DEGs between AP patients and healthy controls

In light of the initial experimental outcomes, it was observed that blood test indicators PT > 12.2, FIB > 3.124, and DD > 0.97 exhibited a strong correlation with SAP, implying potential involvement of genes linked to the coagulation system in the onset and progression of AP. To delve deeper into the underlying mechanisms and key genes in pancreatitis progression, this study selected 67 peripheral whole blood RNA sequencing samples from pancreatitis patients in the Gene Expression Omnibus database, comprising 57 cases of MAP and 10 cases of SAP.

**Figure 3.**
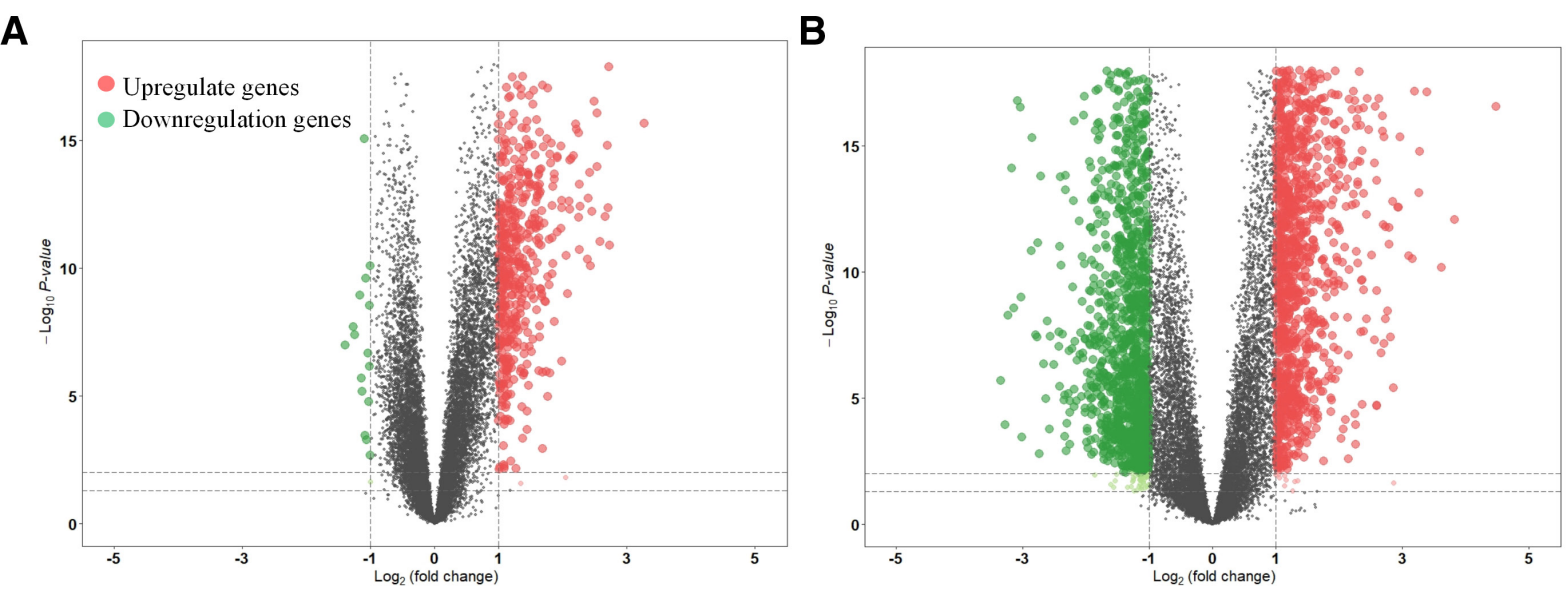
Volcano plot of DEGs between mild, severe acute pancreatitis, and healthy groups: (A) mild, (B) severe. DEGs = differentially expressed genes.

Figure 3A illustrates the comparison between healthy and MAP samples, revealing 514 upregulated genes and 17 downregulated genes. Conversely, Figure 3B showcases the comparison between healthy individuals and SAP patients, uncovering 1732 upregulated genes and 1134 downregulated genes. This trend indicates varying numbers of upregulated and downregulated genes across different severity levels of pancreatitis, suggesting a correlation between the disease’s severity and gene expression alterations. As the severity of AP intensifies, the complexity of gene regulation may also escalate, implying a broader involvement of genes in disease progression, potentially encompassing diverse biological pathways and mechanisms.

Furthermore, we identified the top 10 upregulated and downregulated DEGs between mild and severe pancreatitis alongside normal samples, detailed in Table 5. Subsequent literature review revealed that the top upregulated DEGs, *CD177*,^[15,16]^ and the top downregulated DEGs, *ALOX15*,^[17–20]^ are associated with the coagulation system.

**Table 5:** Top 10 upregulated and downregulated differentially expressed genes in mild acute pancreatitis and severe acute pancreatitis.

	Gene ID	Symbol	*P* value	log2FoldChange
1	383	*ARG1*	3.74E-24	3.78152
2	199675	*MCEMP1*	5.08E-22	3.501546
3	3250	*HPR*	2.18E-16	3.274791
4	6279	*S100A8*	1.22E-23	2.771319
5	306	*ANXA3*	3.01E-21	2.756708
6	6283	*S100A12*	1.53E-21	2.732729
7	57126	*CD177*	1.30E-11	2.730463
8	105372578	*LINC02967*	1.33E-18	2.728421
9	3240	*HP*	4.30E-13	2.709472
10	101927153	*LINC02207*	1.62E-15	2.697491
1	89944	*GLB1L2*	1.07E-07	−1.38943
2	5593	*PRKG2*	2.03E-08	−1.26694
3	1823	*DSC1*	4.18E-08	−1.24579
4	28659	*TRAV24*	1.14E-09	−1.16446
5	105372185	*LOC105372185*	1.99E-06	−1.14075
6	6983	*TRGV9*	6.85E-06	−1.12331
7	107985690	*LOC107985690*	8.88E-16	−1.09796
8	57282	*SLC4A10*	3.67E-04	−1.08579
9	7098	*TLR3*	2.53E-10	−1.07337
10	4897	*NRCAM*	5.32E-04	−1.05714
1	57126	*CD177*	1.09E-44	7.237692
2	4317	*MMP8*	1.49E-43	6.704791
3	131540	*ZDHHC19*	1.12E-43	6.180706
4	6283	*S100A12*	1.68E-57	5.831217
5	55301	*OLAH*	6.10E-30	5.798636
6	199675	*MCEMP1*	1.17E-79	5.752142
7	3586	*IL10*	7.82E-29	5.548932
8	383	*ARG1*	4.90E-42	5.394524
9	5468	*PPARG*	1.23E-24	5.351616
10	8120	*AP3B2*	1.70E-20	5.243791
1	107984706	*LOC107984706*	2.07E-06	−3.35272
2	7503	*XIST*	1.19E-04	−3.2751
3	574407	*OBSCN-AS1*	5.31E-09	−3.23395
4	54674	*LRRN3*	7.97E-15	−3.17837
5	4897	*NRCAM*	2.67E-09	−3.14595
6	9241	*NOG*	1.67E-17	−3.08019
7	8857	*FCGBP*	3.00E-17	−3.03741
8	246	*ALOX15*	1.01E-09	−3.0208
9	9383	*TSIX*	3.54E-04	−3.01273
10	28616	*TRBV4-2*	1.50E-11	−2.865

Green means upregulated differentially expressed genes in mild acute pancreatitis; blue means downregulated differentially expressed genes in mild acute pancreatitis; gray means up-regulated differentially expressed genes in severe acute pancreatitis; purple means downregulated differentially expressed genes in severe acute pancreatitis.

### 3.7. Enrichment analysis of DEGs in AP

**Figure 4.**
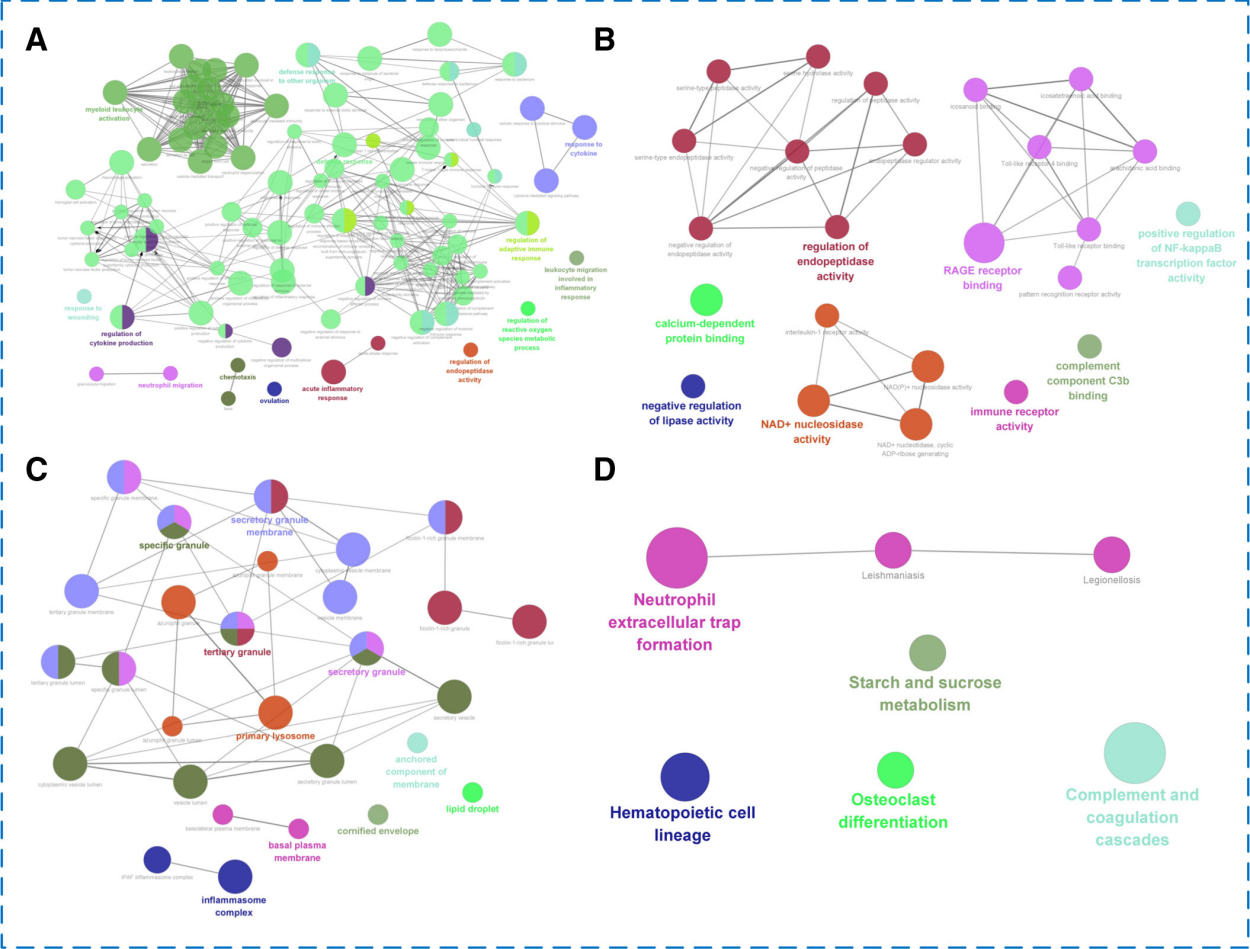
Bioinformatics analysis of DEGs between MAP and the healthy group. (A) Biological processes in GO; (B) molecular functions in GO; (C) cellular components in GO; (D) KEGG. DEGs = differentially expressed genes, GO = gene ontology, KEGG = Kyoto Encyclopedia of Genes, MAP = mild acute pancreatitis.

In the comparison between healthy individuals and those with mild pancreatitis, Figure 4 illustrates that DEGs were predominantly enriched in biological processes associated with acute inflammatory response, regulation of endopeptidase activity, cytokine response, and regulation of cytokine production within the biological process ontology. In the molecular function ontology, these genes were primarily linked to functions such as positive regulation of NF-KappaB transcription factor activation, negative regulation of lipase activation, and Toll-like receptor downstream factor activation. Within the cellular component ontology, these genes showed enrichment in locations like primary lysosomes, inflammasomes, cell membrane components, and inflammatory bodies. Additionally, through KEGG Pathway functional enrichment analysis, further exploration of the potential biological signaling pathways involving these differential genes was conducted. The results highlighted key pathways including neutrophil extracellular trap formation, starch and sucrose metabolism, complement and coagulation cascades, among others. These enrichment analysis findings offer insights for a deeper comprehension of the pathophysiological mechanisms of AP, suggesting that inflammation, immune response, and metabolic dysregulation may play pivotal roles in the initiation of AP.

**Figure 5.**
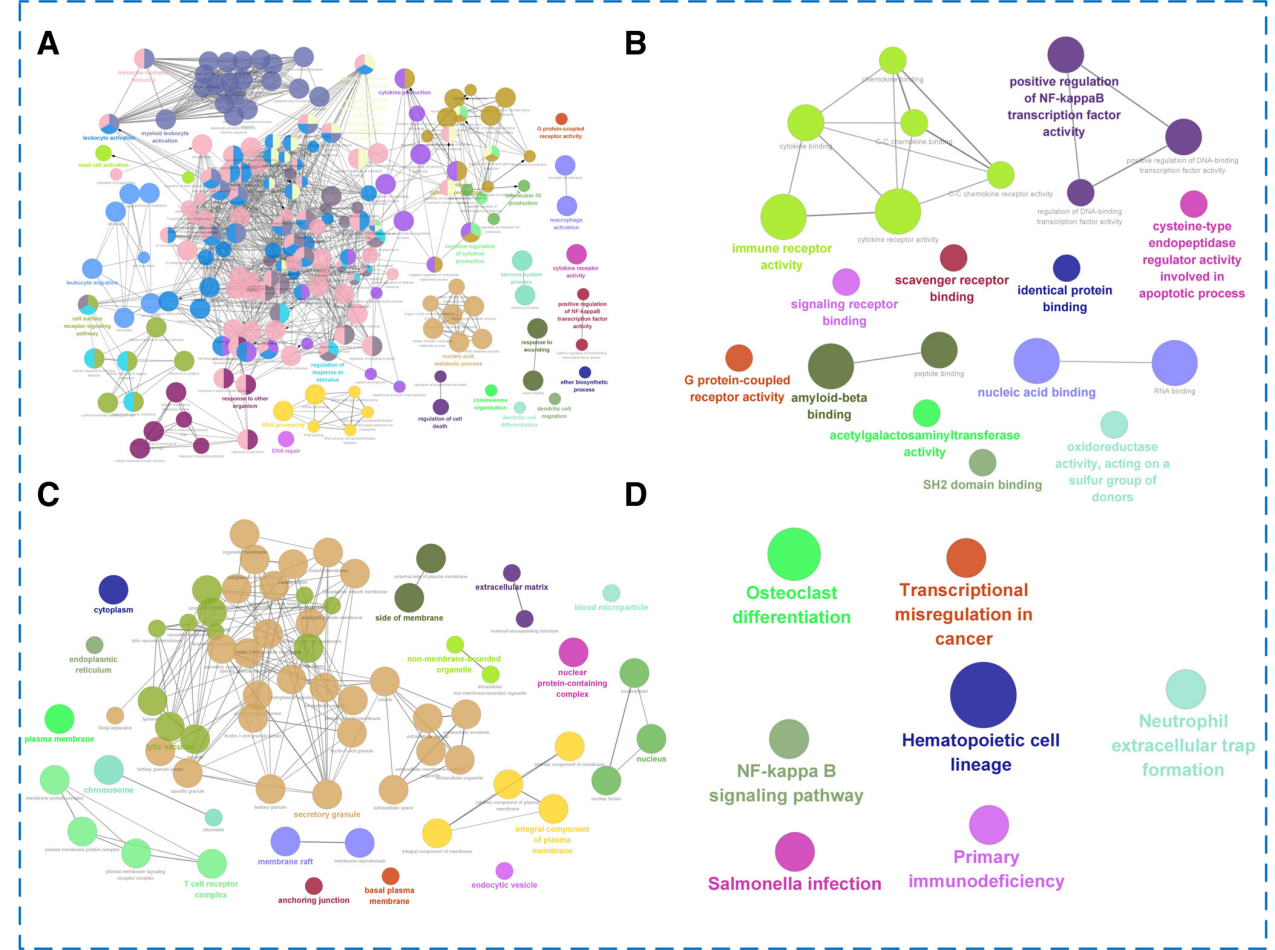
Bioinformatics analysis of DEGs between SAP and the healthy group. (A) Biological processes in GO; (B) molecular functions in GO; (C) cellular components in GO; (D) KEGG. DEGs = differentially expressed genes, GO = gene ontology, SAP = severe acute pancreatitis.

In the comparison between healthy controls and patients with severe pancreatitis, as depicted in Figure 5, it was observed that differentially expressed upregulated and downregulated genes were predominantly enriched in biological processes such as RNA processing, DNA repair, regulation of cell death, nucleic acid metabolism processes, and cell surface receptor signaling pathways. Regarding cellular components, these genes were mainly enriched in the nucleus, cytoplasm, platelets, T-cell receptor complexes, secretory granules, and nuclear protein complexes. In terms of molecular function, these genes were primarily associated with functions like positive regulation of NF-KappaB transcription factor activity, immune receptor activity, nucleic acid binding, and SH2 domain binding. Through KEGG functional enrichment analysis, it was revealed that these differential genes were primarily enriched in processes such as neutrophil extracellular trap formation and primary immunodeficiency. This suggests that severe pancreatitis involves more complex immune responses and inflammatory reactions, leading to extensive cellular damage, dysfunction, and potentially cell death, posing a threat to cell integrity and resulting in severe disruption.

**Figure 6.**
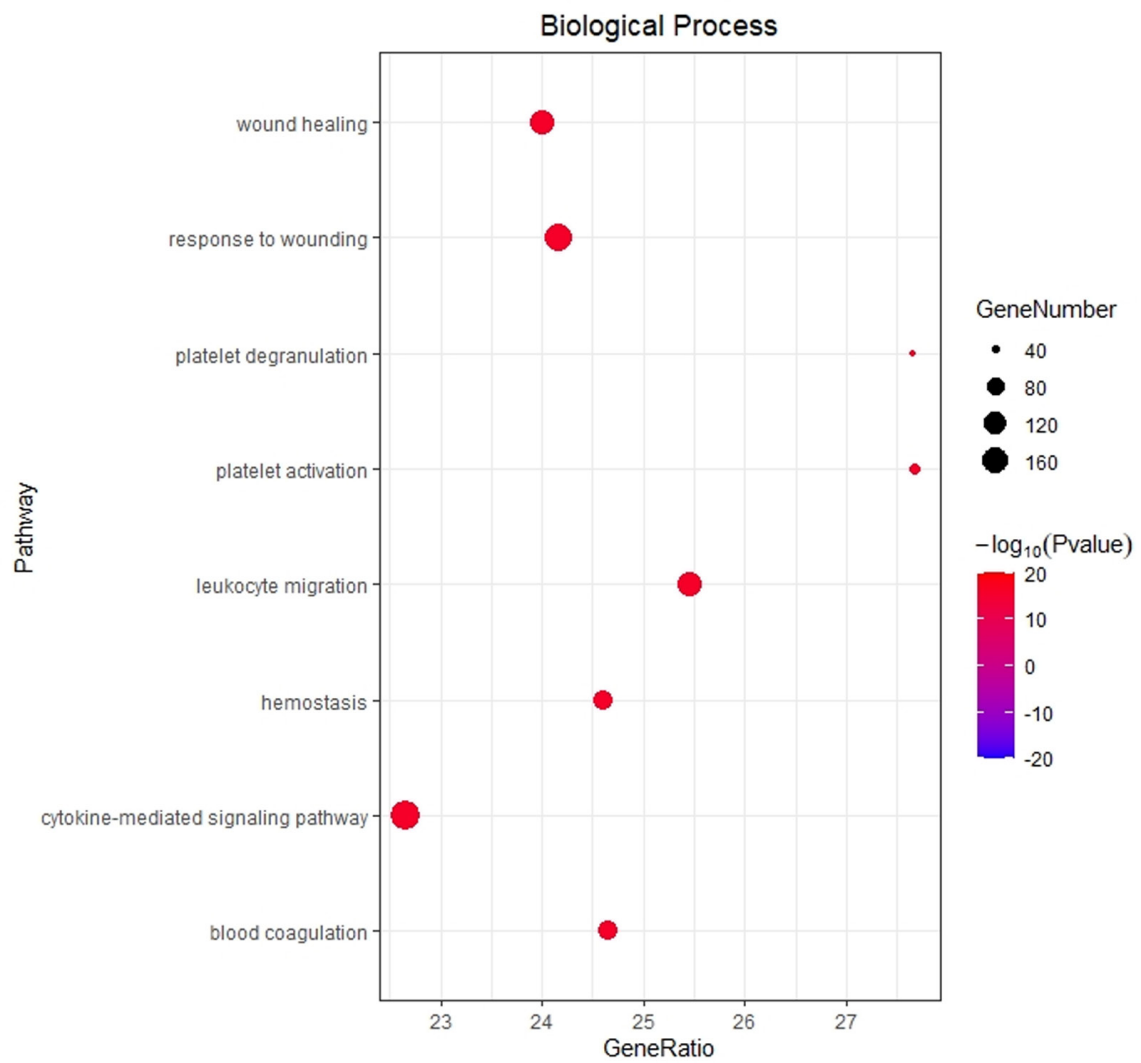
GO analysis of DEGs related to coagulation in biological processes. DEGs = differentially expressed genes, GO = gene ontology.

Following the gene expression profile analysis conducted between healthy controls and patients presenting varying degrees of pancreatitis severity, a set of DEGs associated with the coagulation system was identified. These genes exhibited significant enrichment in biological processes such as blood coagulation, hemostasis, platelet activation and degranulation, leukocyte migration, cytokine signaling pathways, response to injury, and wound healing. These findings suggest that AP, as an inflammatory condition, may impact the expression of pivotal genes involved in these biologically essential processes closely linked to coagulation and inflammation, thereby influencing the status of the coagulation system (refer to Fig. 6).

## 4. Discussion

AP represents a prevalent clinical emergency characterized by sudden onset, rapid progression, and a generally poor prognosis. Recent trends indicate a gradual increase in the incidence of AP, attributed to lifestyle modifications and dietary habits. Reports highlight an overall mortality rate ranging from approximately 2% to 10% for AP cases, with SAP presenting a significantly higher mortality rate, reaching up to 30%.^[3]^ Hence, alongside enhancing SAP treatment modalities, it is crucial from a clinical perspective to actively pursue or establish a scientific, rational, and straightforward evaluation framework to precisely assess patient conditions and prognoses.

The primary etiologies of AP encompass pro-inflammatory factors, biliary origin, fasting, alcohol-induced injury, and pancreatic stones. These factors may trigger excessive pancreatic enzyme secretion, culminating in pancreatic injury and subsequent AP development.^[21]^ Research indicates that SAP incidence is notably correlated with elevated serum levels of lipase, amylase, C-reactive protein, coagulation factors, white blood cells, and neutrophils compared to mild AP cases.^[22–24]^

In AP, the production of inflammatory factors and endotoxins triggers a cascade leading to the excessive release and overexpression of tissue factors. This process activates both exogenous and endogenous coagulation pathways, disrupting the delicate balance between coagulation and FIB. The consequent consumption of various coagulation factors results in a notable prolongation of PT and APTT, along with increased FIB and elevated levels of FIB and DD. These changes culminate in systemic Disseminated Intravascular Coagulation and expedite the progression to SAP.

Our study revealed significantly higher PT, FIB, and DD levels in the SAP group compared with those in the mild AP group, indicating a robust correlation between AP severity and the values of these 3 markers. Subsequently, we incorporated gender, age, APTT, PT, FIB, and DD into a multifactor logistic regression model. The outcomes demonstrated that, holding other variables constant, each unit increase in PT and DD corresponded to a 3.456- and 2.197-times higher risk of SAP occurrence, respectively. This highlights PT and DD as independent factors influencing the assessment of AP severity, unaffected by variations in gender, age, APTT, or FIB levels.

Our findings not only establish PT and DD as significant risk indicators for SAP but also quantify the relationship between changes in these parameters and the heightened risk of SAP development. This quantification enhances the accuracy and reliability of clinical prognostications in AP management.

Common traditional scoring systems for AP currently in use include the APACHE II scoring system,^[11]^ Ranson scoring system,^[12]^ and CTSI scoring system.^[13]^ The APACHE II system, known for its complexity, boasts high accuracy and predictive value in assessing the progression and severity of AP. A score of ≥8 serves as an assessment threshold for SAP, showing a sensitivity range of 65% to 83%, a specificity range of 77% to 91%, a positive predictive value of 23% to 69%, and a negative predictive value of 86% to 99%.^[25]^

The Ranson scoring system stands as an objective prognostic tool tailored for early-stage AP severity evaluation among patients. Although it may not encompass physiological functions as extensively as APACHE II, it effectively delineates local lesions in AP, compensating for the limitations of the APACHE II system. However, the Ranson scoring necessitates evaluation after 48 hours from the onset, thereby impeding early monitoring within the initial 48 hours of disease onset.^[13]^

The CTSI scoring system is revered as the gold standard for diagnosing and assessing AP severity. Nonetheless, it primarily observes morphological alterations in the pancreas and may not accurately mirror physiological changes within the body. These scoring systems exhibit certain drawbacks such as complexity, delayed establishment, and susceptibility to influences from treatment modalities.^[14]^

PT monitors the extrinsic pathway, whereas APTT tracks the intrinsic pathway, enabling the dynamic assessment of coagulation function to elucidate the body’s condition progression during AP.^[26]^ FIB, a crucial clotting factor, swiftly reacts and participates in platelet aggregation and thrombus formation during AP.^[27]^ DD serves as a marker of FIB, reflecting the biological attributes of active clotting and fibrinolytic enzymes, exhibiting significant elevation during excessive FIB.^[28]^ These laboratory coagulation indicators are characterized by their simplicity, sensitivity, and reliability.

This study employed the commonly utilized APACHE II, Ranson, and CTSI scoring systems as experimental controls to evaluate the significance of peripheral blood coagulation indicators and DD values in the early diagnosis of SAP and MAP. The research revealed that the mean scores of APACHE II, Ranson, and CTSI in the SAP group markedly exceeded those in the mild AP group. Subsequent correlation analyses unveiled a positive association between APACHE II and Ranson scores with PT, FIB, and DD, alongside a positive correlation between the CTSI score and PT. These findings suggest that PT, FIB, and DD can function as assessment metrics for the early diagnosis of AP, akin to the APACHE II, Ranson, and CTSI scoring systems.

Presently, there exists a lack of quantified criteria for serological indicators to differentiate between MAP and SAP cases. To address this gap, the study performed ROC curve analysis on PT, FIB, and DD to ascertain their value in assessing and distinguishing between the SAP and MAP groups. The outcomes indicated that instances where PT > 12.2, FIB > 3.124, and DD > 0.97 were associated with an elevated likelihood of SAP development, underscoring the utility of PT, FIB, and DD as correlated markers for the assessment of SAP.

The preceding clinical study highlighted in this paper identified PT, FIB, and DD as markers significantly correlated with SAP. However, the underlying mechanisms remain unclear. Through bioinformatics analysis, this study unveiled a positive correlation between AP severity and the number of DEGs, alongside a positive correlation with gene regulatory complexity.

Gene ontology and Kyoto Encyclopedia of Genes and Genomes enrichment analyses indicated that AP could induce alterations in gene expression associated with blood coagulation and hemostasis, influencing clotting factor levels and activity. Platelet activation and degranulation processes may also be impacted. Changes in genes related to leukocyte migration and cytokine-mediated signal transduction could modify immune cell activity and migration capabilities, influencing inflammation severity and immune responses, indirectly affecting coagulation status.

Among the DEGs, the study focused on the *CD177* gene and *ALOX15* gene associated with the coagulation system. CD177, a neutrophil marker, is exclusively expressed in neutrophils, immature neutrophilic precursors, and bone marrow cells.^[14]^ Acting as a binding partner for platelet endothelial cell adhesion molecule-1, CD177 inhibits platelet activation pathways mediated by ADP, cross-linked collagen peptides, and thrombin when PECAM-1 is activated in vitro.^[16]^

Due to CD177’s high affinity for PECAM-1 and its functional reliance on this interaction, CD177 plays a crucial role in neutrophil migration through endothelial cells. Consequently, activated neutrophils binding to endothelial cells and platelets during initial injury stages impede blood flow in vessels.^[29]^ Studies have demonstrated a positive correlation between CD177 and AP severity. Thus, this study proposes that *CD177*, through its regulation of PECAM-1, influences coagulation function in pancreatitis.^[30]^

ALOX15, a member of the lipoxygenase family, plays a pivotal role in catalyzing the oxidation of various fatty acids to generate multiple lipid components, thereby contributing to the pathophysiological processes of diverse immune and inflammatory diseases.^[17–19]^ ALOX15’s involvement has been established across a spectrum of conditions, including liver diseases, cardiovascular disorders, cerebrovascular ailments, and diabetes.^[31]^

Prior research has highlighted the association of ALOX15 with various diseases. Specifically, the ALOX15/15-HETE pathway has been identified as capable of stimulating platelet activation and enhancing thrombin generation.^[19]^ As a result, there is a hypothesis that modulation of coagulation function through the ALOX15/15-HETE signaling pathway could impact the onset and progression of AP.

## 5. Conclusion

This study categorized AP patients into 2 groups based on samples and assessed their levels of PT, APTT, FIB, and DD. Spearman linear correlation analysis was applied to PT, APTT, FIB, and DD in relation to APACHE II score, Ranson score, and CTSI score. Multiple logistic regression analysis was utilized to identify the risk factors influencing the severity of AP. Moreover, bioinformatics methods were employed to scrutinize the DEGs and the pathological processes involving these genes, delving into potential mechanisms and key genes in AP progression. The primary conclusions drawn from this study are as follows:

Elevated levels of PT (>12.2 seconds), FIB (>3.124 g/L), and DD (>0.97 mg/L) demonstrate a strong correlation with the progression of AP and can function as important clinical markers for severity assessment. This phenomenon may be attributed to variations in gene expression related to blood coagulation and hemostasis induced by AP, subsequently impacting the levels and activity of coagulation factors. DEGs *CD177* and *ALOX15* potentially play a role in the onset and advancement of AP by modulating the coagulation system. Monitoring PT, FIB, and DD levels alongside differential gene expression data in AP can offer clinicians a more precise evaluation of the condition, enabling the accurate assessment of disease progression and the development of personalized treatment strategies to enhance treatment efficacy and safety. In addition, the regulatory influence of DEGs in these biological processes in AP provides insights into the molecular and cellular mechanisms of pancreatitis, presenting potential targets for diagnosis and treatment enhancement.

## Author contributions

**Conceptualization:** Mengqi Hong, Wenyi Hu.

**Data curation:** Hongda Zhu, Mengqi Hong, Wenyi Hu, Qiyan Yu.

**Methodology:** Hongda Zhu, Mengqi Hong.

**Software:** Hongda Zhu, Qiyan Yu.

**Visualization:** Hongda Zhu.

**Funding acquisition:** Mengqi Hong, Wenyi Hu.

**Formal analysis:** Qiyan Yu.

**Resources:** Qiyan Yu.

**Writing – original draft:** Hongda Zhu, Mengqi Hong.

**Writing – review & editing:** Hongda Zhu, Wenyi Hu.
